# Effects of *In Ovo* Methionine-Cysteine Injection on Embryonic Development, Antioxidant Status, IGF-I and TLR4 Gene Expression, and Jejunum Histomorphometry in Newly Hatched Broiler Chicks Exposed to Heat Stress during Incubation

**DOI:** 10.3390/ani9010025

**Published:** 2019-01-12

**Authors:** Hamada A. M. Elwan, Shaaban S. Elnesr, Qianqian Xu, Chao Xie, Xinyang Dong, Xiaoting Zou

**Affiliations:** 1 College of Animal Science, Zhejiang University, Hangzhou 310058, China; hamadaelwan83@mu.edu.eg (H.A.M.E.); ssn00@fayoum.edu.eg (S.S.E.); qianqianxu@zju.edu.cn (Q.X.); chao-xie@zju.edu.cn (C.X.); sophiedxy@163.com (X.D.); 2 Animal and Poultry Production Department, Faculty of Agriculture, Minia University, El-Minya 61519, Egypt; 3 Poultry Production Department, Faculty of Agriculture, Fayoum University, Fayoum 63514, Egypt

**Keywords:** Methionine-Cysteine, *In ovo* injection, IGF-I, Glutathione, HSP90

## Abstract

**Simple Summary:**

In the current study, we hypothesize that the *In ovo* concurrent injection of methionine (Met) and cysteine (Cys) may have positive effects on embryonic development, insulin-like growth factor-I (IGF-I) and toll-like receptor-4 (TLR4) gene expression, antioxidant status, biochemical profile, and jejunum histomorphometry of newly hatched broiler chicks exposed to heat stress during the incubation period. As indicated, the *In ovo* injection of Met and Cys resulted in increased hatch weight, higher levels of total antioxidant capacity, and glutathione activity in the serum and tissues. At the same time, a decrease in the level of heat shock protein-90 and an increase in TLR4 and IGF-I expression in the tissues was observed. In addition, the *In ovo* injection of Met and Cys improved jejunum histomorphometric parameters of newly hatched broiler chicks. Generally, the concurrent *In ovo* injection of Met and Cys improved broiler embryonic development, antioxidant status, TLR4, and IGF-I expression and jejunum histomorphometric parameters in newly hatched broiler chicks exposed to heat stress conditions during incubation.

**Abstract:**

Sulfur amino acids are typically the first-limiting amino acids (AA) used in protein metabolism in poultry. Therefore, we hypothesized that their utilization in the pre-hatch period would affect embryonic development, IGF-I and TLR4 gene expression, antioxidant status, serum biochemical profile, and jejunum histomorphometry of newly hatched Ross broiler chicks incubated under heat stress conditions. A total of 150 fertile broiler eggs were subjected to heat stress (39.6 °C for 6 h/d) from d10 until d18 and injected at d 17.5 of incubation with methionine and cysteine (Met-Cys) at a dose of 5.90 mg l-methionine plus 3.40 mg l-cysteine. The effects of Met-Cys administration were examined and compared with the control (Non-injected group) and 0.75% NaCl injected group. The results showed that no significant differences among all groups in serum protein profiles (total protein, albumin, globulin, and albumin/globulin ratio) and creatine kinase were observed. The level of heat shock protein-90 was decreased with Met-Cys *In ovo* injection. The *In ovo* injection of Met-Cys also improved the values of total antioxidants capacity and glutathione in examined tissues. At the same time, an increase in fold change mRNA abundance of IGF-I and TLR4 was observed after Met-Cys injection in tested tissues. Finally, an increase of 29% in villus area was found after Met-Cys injection compared to the control group. In conclusion, the *In ovo* injection of Met-Cys resulted in improved embryonic development, IGF-I and TLR4 gene expression, antioxidant status and jejunum histomorphometry of newly hatched broiler chicks exposed to heat stress during incubation.

## 1. Introduction

*In ovo* injection of amino acids (AA) could be utilized due to its positive effects on several physiological and biochemical parameters, including improvement of oxidative status, key adapting function of metabolic processes during early development [1]. Several factors affect avian embryogenesis, such as *In ovo* nutrients injection [2], and temperature [3]. There are discrepancies in the existing literature concerning the incubation temperatures and exposure periods, which lead to heat stress [4]. Previous studies have documented that slight alterations in the incubation temperature could affect avian embryogenesis and metabolism [5], small intestine development [6], improve thermo-tolerance through the ability of broiler chicks to survive and overcome stress, due to excessive heat exposure [7,8].

Among sulfur amino acids (SAA), Methionine (Met) is typically the first-limiting AA for protein requirement in poultry diets [9]. Methionine is considered to play main roles in the body. These roles are, such as protein synthesis, a glutathione precursor, reactive oxygen species (ROS) elimination and methylation reaction of DNA [10]. Cysteine serves as a semi-essential AA because it can be synthesized from methionine and serine by trans-sulfuration [11]. Recently, Han et al. [12] found that AA levels, including leucine, were significantly declined as an effect of thermal treatment on the embryonic brain and liver. It was also reported that heat stress could affect SAA metabolism in chicks [13]. Many studies demonstrate an increased protein synthesis rate within the pre-hatch growth period as a result of the *In ovo* AA injection [14,15]. It is further shown that pre-hatch embryo growth is affected by the *In ovo* injected substance. For example, *In ovo* injection of β-hydroxy-β-methyl butyrate and carbohydrates at days 16, 17, and 18 of incubation period led to an improvement in digestive track development, digestion capacity and nutrients metabolism, muscle development, and breast meat production [16]. At the late term of incubation avian embryos grow faster, so their nutrient requirements increase. Since the nutrients of eggs are not enough [16], exogenous AA could be *In ovo* applied [17]. Many types of research have assessed different treatments during the pre- and immediate post-hatch periods with the intention to improve the growth rate and body weight. Previous trials suggested that chick weight at hatch is correlated with the weight at the end of commercial production [18]. Uni et al. [19] demonstrated that late-term (16–18 day of incubation) *In ovo* injection of supplemented solutions containing carbohydrates led to a 5–6% increase in body weight of the newborn chicks and this difference continued till the last day of the examined period of that study (25 days of age). Moreover, Chowdhury et al. [20] reported that AA plays critical roles in the growth and controlling body temperature.

However, the effect of *In ovo* injection of SAA during the incubation period under heat stress conditions on the embryo development and hatching quality are not known [21]. Thus, further research related to the *In ovo* injection is necessary. Therefore, the purpose of study was to assess the effects of the late *In ovo* injection of methionine-cysteine (Met-Cys) at a dose of 5.90 mg l-methionine plus 3.40 mg l-cysteine on embryonic development, insulin like growth factor-I (IGF-I) and toll like receptor-4 (TLR4) gene expression, antioxidant status, serum biochemical profile, and jejunum histomorphometry in newly hatched broiler chicks exposed to heat stress during incubation. 

## 2. Materials and Methods

The experimental protocol utilized in this research were complied with the Chinese guidelines for animal welfare and approved by the Animal Care and Use Committee of the Animal Science College of Zhejiang University (No. ZJU2013105002), Hangzhou, China.

### 2.1. Eggs and Incubation Conditions

Fertile broiler eggs (Ross 308) were obtained from a commercial breeder flock (Hangzhou, Zhejiang, China). Eggs were transferred into an incubator (Capacity 176 eggs, Model ZF440, Zhengda Incubation Equipment Co. Ltd., Dezhou, China), automatic egg turning every 2 h. Eggs were incubated under optimal incubation conditions (37.8 °C and 50% relative humidity) from d 1 to 10 and then exposed to high temperature (39.6 °C) for 6 h daily (10:00–16:00) from d 10 until d 18 of the incubation period. On d 10 of embryonic development, eggs were candled with a lamp, and those containing dead embryos were removed from the incubator. After the 18th day of the incubation, eggs were transferred from the setter tray to the hatcher basket, the incubation temperature was 37.5 °C and relative humidity was 65–70%.

### 2.2. Methionine-Cysteine In ovo Injection

l-methionine (C_5_H_11_NO_2S_) and l-cysteine (C_3_H_7_NO_2S_), (purity ≥ 99%) were purchased from Beijing Solarbio Sciences and Technology Co., Ltd. (Beijing, China). At d 17.5 of incubation, eggs (*n* = 150) were randomly distributed into three groups of 50 eggs with a similar mean weight using AB204-N scale (METTLER TOLEDO instruments, Shanghai, Co., LTD, China), 0.0001 measurement accuracy. The first group consisted of non-injected eggs and served as control, the second group was only injected with 1.0 mL of 0.75% saline solution (NaCl), and the third group was injected with a freshly prepared solution of a mixture of 5.90 mg l-methionine plus 3.40 mg l-cysteine dissolved in 1.0 mL saline solution (Met-Cys). This concentration was calculated as 2% of the content of methionine and cysteine (295 and 170 mg/egg, respectively) in the egg according to Ohta et al. [14] and Bhanja et al. [22].

Eggs were injected through the air cell using a 21-gauge needle, having a short beveled tip to target the amnion. Before injection time, pilot tests with visible dye confirmed the safe delivery of solutions into the amnion [23].

### 2.3. Embryonic Development Index

On the day of hatch, the hatchability was expressed as a percentage of fertile eggs. Body weight of chicks and hatching time (the number of hours between setting eggs in the incubator until hatching) were recorded. Ten chicks per group were slaughtered to record the weight of liver, heart, pectoral muscles, small intestine, left lung, and residual yolk and then expressed as % of the chick weight.

### 2.4. Serum Biochemical Indicators

Ten blood samples from each group at hatching time were randomly collected after slaughtering and then were centrifuged for 10 min (3000× *g*) at room temperature to separate the serum that was stored in Eppendorf tubes (1.5 mL) at −80 °C until analyzed. Serum concentrations of total protein, albumin, uric acid, and creatine kinase were measured using an automated system (7020 analyzers, Hitachi High-Technologies Co., Tokyo, Japan). Thyroid hormones in serum (triiodothyronine (T3), thyroxine (T4)) and heat shock protein-90 (HSP90) were detected by using ELISA kits. These previous parameters were determined according to the instructions of kits (Nanjing Jiancheng Bioengineering Institute, Nanjing, China).

### 2.5. Jejunum Histomorphometry

Immediately after slaughter, nine Jejunum samples (three specimens each of about 1 cm from three locations; after the duodenal loop, in the medial and before Meckel’s diverticulum) from each group (three chicks per group) were collected and prepared. The samples were washed in physiological saline solution, and fixed in 10% buffered formalin. To evaluate Jejunum histomorphology, hematoxylin-eosin-stained was used. To determine villus height (VH), width (VW), area, width and depth of crypt and jejunum muscular thickness (JMT), 15 vertical villi were evaluated on each section in all three groups using image processing and analysis system of the software, AxioVision, specialized for the microscope. The villus height was measured from the top of the villus to the top of the lamina propria. The villus surface area was calculated by the formula 2π × VH × (VW/2), where VH is villus height and VW is villus width. Also, histological absorptive surface amplification (HASA) was calculated according to Kisielinski et al. [24] as follows:

$$\mathrm{HASA}=\frac{\left(\mathrm{Villus}\;\mathrm{width}\;\times \;\mathrm{Villus}\;\mathrm{height}\right)+\left(\mathrm{Villus}\;\mathrm{width}/2\;+\;\mathrm{Crypt}\;\mathrm{width}/2\right)2-\left(\mathrm{Villus}\;\mathrm{width}/2\right)2}{\left(\mathrm{Villus}\;\mathrm{width}/2+\;\mathrm{Crypt}\;\mathrm{width}/2\right)2}$$

### 2.6. Assay of Antioxidant Biomarkers in the Tissues and Serum

Total antioxidant capacity (TAOC), glutathione (GSH) in serum and tissues (liver, heart, pectoral muscle, kidney, and small intestine) in ten samples/group were determined using Nanjing Jiancheng Bioengineering Institute (Nanjing, China) reagent kits. The tissue samples were stored at −80 °C, then homogenized with 10 volumes (0.1 g per mL) using isotonic physiological saline by a Tissuelyser-24 (Xin Jin Technology, Shanghai, China) at 65 Hz for 60 s, and instantly centrifuged at 15,294.24 × *g* at 4 °C for 10 min. The supernatant was collected for further analyses.

### 2.7. RNA Isolation and cDNA Synthesis

From approximately 50 mg of tissue, total RNA was isolated by using TRIzol reagent kits (Invitrogen, Carlsbad, CA, USA) and other reagents according to the manufacturer’s instructions. The purity of RNA was determined using Nanodrop 2000 UV-Vis spectrophotometer (Thermo-fisher scientific, Wilmington, MA, USA) at an optical ratio of OD260/OD280, which was in the range of 1.9 and 2.1. The RNA quality was inspected by agarose 1.2% gel electrophoresis. The cDNA was synthesized by reverse transcription by using HIScript IIQRT SuperMix for qPCR (Vazyme, Nanjing, China) using the protocol of the manufacturer.

### 2.8. Quantitative Real-Time Reverse Transcription-PCR

The gene expressions of IGF-I and TLR4 in heart and pectoral muscles, jejunum and liver tissues of newly hatched broiler chicks were quantified by quantitative real-time PCR system (ABI 7500, Applied Biosystems, Foster City, CA) according to the protocol of ChamQTM Universal SYBR qPCR Master Mix (Vazume, Nanjing, China). Gene-specific primers of IGF-I, TLR4 and reference gene (18 s) were as follows: IGF-1-F: GTATGTGGAGACAGAGGCTTC; IGF-1-R: TTTGGCATATCAGTGTGGCGC [25]; TLR4-F: ATGCCCAGCAGAGCGGCTCCCA; TLR4-R: CTTGATAGCTGCCTGGAGGAAGGCAATC A [26]; 18S-F: ATTCCGATAACGAACGAGACT; 18S-R: GGACATCTAAGGGCATCACA [27]. The PCR reaction was performed within the following thermal protocol: 95 °C/30 s, followed by 40 cycles of 95 °C/10 s and 60 °C/30 s, then followed by 95 °C/15 s, 60 °C/60 s and 95 °C/15 s. There were 6 samples per group, each sample was conducted in duplicate, and no template control was included. The mRNA levels were standardized as the ratio to 18S rRNA in arbitrary units by using the ^2−ΔΔ^Ct methods [28].

### 2.9. Statistical Analysis

Treatment-dependent changes were analyzed by ANOVA. Statistical differences among means were considered significant at *p* ≤ 0.05. A posthoc test (Tukey-Kramer) was performed following ANOVA. JMP version 6.0 (SAS Institute, Cary, NC, USA) was used for all analyses. Data are presented as means and pooled SEM.

## 3. Results

### 3.1. Embryonic Development Parameters

The effects of *In ovo* injection of Met-Cys on embryonic development of newly hatched broiler chicks exposed to heat stress conditions are illustrated in Table 1. Hatchability of fertile eggs was 78%, 78% and 80% for the control, NaCl and Met-Cys injected groups, respectively, with no significant (*p* = 0.758) differences among all groups. Concerning to hatch time, NaCl and Met-Cys injected groups recorded longer (*p* < 0.001) hatching time compared with the control group. Moreover, *In ovo* injection of Met-Cys led to an increase of chick weight compared to the other groups (*p* = 0.027). On the other hand, the relative weight of the liver, heart, pectoral muscle, small intestine, left lung, and the residual yolk was not affected by the *In ovo* injection.

### 3.2. Serum Biochemical Parameters

The effects of *In ovo* Met-Cys injection on serum biochemical parameters are listed in Table 2. There were no significant differences among all groups in serum total protein profiles (total protein, albumin, globulin, and albumin/globulin ratio) and creatine kinase. In contrast, levels of serum triiodothyronine, thyroxin, and uric acid were higher (*p* < 0.01) in Met-Cys group compared with other groups. Moreover, levels of serum HSP90 were 19.87% lower in Met-Cys injected than the control group.

### 3.3. Antioxidant Biomarkers

Sulfur amino acids (Met-Cys) appeared to enhance antioxidant enzyme activities (Table 3). Total antioxidant capacity (TAOC) and glutathione (GSH) levels of newly hatched broiler chicks either in serum or in tissues were higher (*p* < 0.05) in the In ovo Met-Cys injected group compared to the other groups. As indicated, TAOC of Met-Cys group was increased by 30.94, 17.94, 11.86, 13.01, 56.95, and 38.42% compared to the control group in serum, liver, small intestine, kidney, heart and pectoral muscle, respectively. In addition, GSH had also greater values in Meth-Cys injected group; 56.88, 10.49, 8.06, 27.17, 27.22 and 20.35% compared to controls in serum, liver, small intestine, kidney, heart, and pectoral muscle, respectively.

### 3.4. IGF-I and TLR-4 mRNA Relative Gene Expression

**Figure 1:**
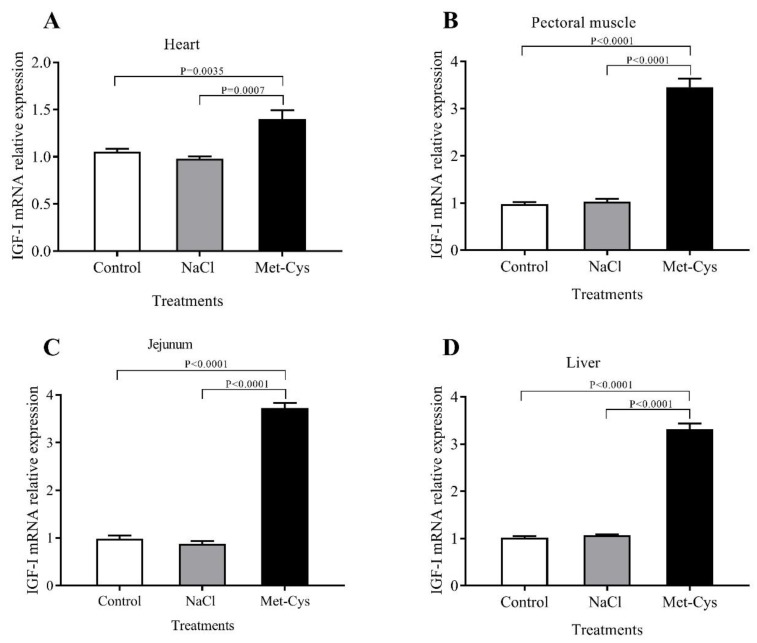
Effect of Met-Cys *In ovo* injection on mRNA relative gene expression of insulin-like growth factor (IGF-I) in the heart, pectoral muscle, jejunum and liver tissues of day-old broiler chicks, (**A**–**D**). Control = non-injected; NaCl = injected with 0.75% NaCl; Met-Cys = injected with 5.9 mg l-methionine plus 3.4 mg l-cysteine.

**Figure 2:**
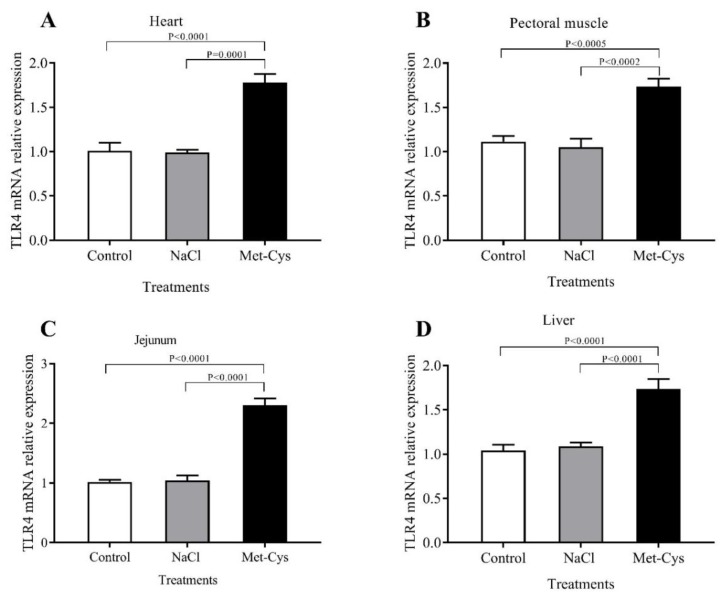
Effect of Met-Cys *In ovo* injection on mRNA relative gene expression of chicken toll-like receptor 4 (TLR4), in the heart, pectoral muscle, jejunum and liver tissues of day-old broiler chicks, (**A**–**D**). Control = non-injected NaCl = injected with 0.75% NaCl; Met-Cys = injected with 5.9 mg l-methionine plus 3.4 mg l-cysteine.

Effects of *In ovo* Met-Cys injection on IGF-I and TLR-4 gene expression of heart, pectoral muscle, jejunum, and liver are presented in Figure 1 and Figure 2. The In ovo injection of Met-Cys significantly (*p* < 0.01) increased the fold change of IGF-I mRNA relative gene expression in heart, pectoral muscle, jejunum or liver (Figure 1). However, the IGF-I Ct ratio showed no significant differences between the NaCl and the control group. Concerning the relative expression of the TLR-4 gene in heart, pectoral muscle, jejunum or liver, mRNA abundance was significantly higher in the Met-Cys group compared to the other groups (Figure 2).

### 3.5. Jejunum Histomorphometric Analysis

**Figure 3:**
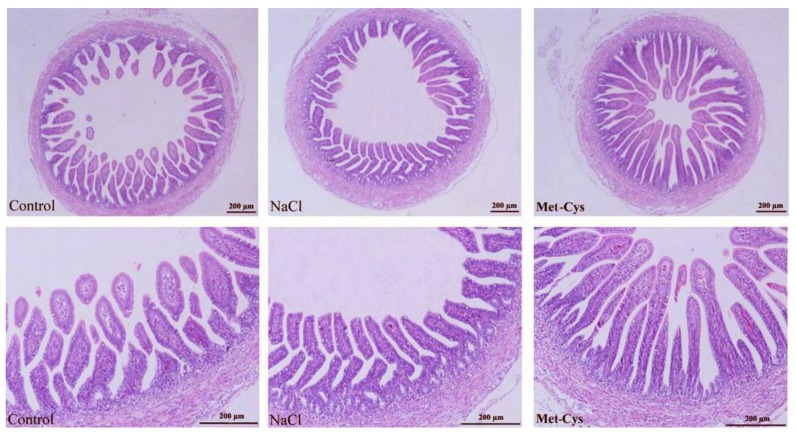
Photomicrographs of intestinal villi from the jejunum of broiler chicks on the day of the hatch in control, NaCl and Met-Cys groups. Control = non-injected NaCl = injected with 0.75% NaCl; Met-Cys = injected with 5.9 mg l-methionine plus 3.4 mg l-cysteine.

Significant differences were observed in the jejunum histological structure as an effect of *In-ovo* Met-Cys injection (Table 4). Histological assessment of the jejunum sections showed larger villus height, width, area and crypt depth in *In-ovo* Met-Cys injected compared to other groups. However, crypt width was not significantly (*p* = 0.199) different in Met-Cys compared to the other groups, whereas HASA and JMT had increased values in Met-Cys group (*p* < 0.001) compared with other groups. Villus height was more uniform in Met-Cys group than the other groups that contained a non-uniform pattern in the villus height (Figure 3). 

## 4. Discussion

In the current study, 6 h of daily exposure to heat stress (39.6 °C) between the 10th and 18th day of incubation were imposed based on the hypothesis that thermal stress induces more intense effects during the critical time window of the hypothalamus-hypophysis-adrenal or/and -thyroid axis development [29]. In this study, Met-Cys injection improved embryonic development and resulted in heavier chicks, due to the longer hatching time. At the same time, the increased thyroid hormone values are associated with improved embryo metabolism. Many studies have shown that AA quantity in the egg is only sufficient until hatching [30]. The application of *In-ovo* injection can lead to an improvement of chick’s weight at hatching [18]. A previous study of Nazem et al. [15] also indicated that *In-ovo* injection of methionine increased the weight at hatch, and this may be attributed to the improved antioxidant status of the embryos. In the current study, the use of Met-Cys led to an increase in hatching weight. Also, Uni and Ferket [31] pointed out that *In ovo* injection of amino acids increased the weight at hatch. As shown in the present study, the injection of Met-Cys mixture led to a decrease of HSp90 levels and this may be due to enhancing the antioxidant status of the embryos. It has been suggested that embryos are more sensitive to temperatures above 37.8 °C [32]. Also, continuous thermal manipulation negatively affects hatchability and performance of embryos exposed to heat stress (39.5 °C) from day 7 to 16 of the embryonic period. Our results indicated that there were no significant differences in residual yolk among groups and these results are in agreement with that obtained by Barri et al. [6] who suggested that no significant differences were observed in the yolk weights of chicks as a result of alterations in the incubation temperature. Moreover, Yahav et al. [33] concluded that increasing incubation temperature to 38.5 °C for 3 h daily from 16 to 18 days of incubation had a positive effect on thermoregulation by causing a reduction in plasma thyroid hormone concentrations. *In ovo* injection of Met-Cys after heat stress treatment led to an increase in plasma thyroid hormone concentrations (T3 and T4). This result agreed with those of Lu et al. [34] who found that levels of thyroid hormones were positively correlated with chick embryonic weight and they suggested that thyroid hormones appear to be critically important in maintaining normal growth and development during chick embryogenesis.

To assess the effects of *In ovo* injection of Met-Cys on oxidation biomarkers, TAOC and GSH parameters were measured in serum and different tissues of newly hatched broiler chicks. Met-Cys group had greater amounts of TAOC and GSH in the serum, liver, small intestine, kidney, heart, and pectoral muscle compared to the control group. Nevertheless, GSH acts not only as a cellular antioxidant, but also as storage for extra cysteine within the cell [35]. It is well known that heat stress led to increasing H_2_O_2_ via induced oxidative stress, which affects the capacity of different antioxidant enzymes [36]. Hydrogen peroxide is known as a trigger of GSH production [37]. Shen [38] reported that there were increased GSH levels in the duodenal mucosa of chicks fed a diet supplemented with 0.285% L-Met, which demonstrated that a greater portion of L-Met shifts to GSH production in the gastrointestinal tract. Moreover, Met-Cys are efficient ROS scavengers under physiological conditions [38]. The Met-Cys residues in proteins can react readily with ROS and form i.e., methionine sulfoxide, which can be reduced by methionine sulfoxide reductases to methionine [39]. According to Surai [40], there are many factors that can interfere with the antioxidant status of newly hatching chicks, including temperature, humidity, carbon dioxide fluctuations, vaccination, delay in collecting chicks from the incubator, disease challenges and hatching time. Incubation temperature could affect embryonic development, oxidation, and phosphorylation in tissues, leading to the excessive production of ROS [41]. The ROS scavengers, cysteine, and glutathione (GSH) are direct products in the transsulfuration pathway, which can alleviate the deleterious effect of lipid and protein oxidation in the cells [42]. Amino acids are the main source of energy for the liver [43]. Under heat stress conditions, most of the amino acids are reduced, apart from cysteine that had increased values [44]. An increased concentration of Cys is consistent with the observed reduced glutathione levels under heat stress. Since Met metabolism affects oxidative status, it is reasonable to hypothesize that dietary Met-Cys supplementation may play a beneficial role in alleviating oxidative stress. *In ovo* injection of Met-Cys mixture led to an increase in GSH levels that result in reduced values of H_2_O_2_ and lipid peroxides [35]. Cellular GSH content is influenced by dietary SAA [45]. A previous investigation reported that dietary Met supplementation was associated with an enhanced GSH synthesis in broilers [46]. In addition, protein carbonyl content is the most commonly used marker of protein oxidation [47]. This result may be related to methionine (Met-Cys), which serves as a precursor for cysteine (Cys), and GSH, [48,49]. Thus, *In ovo* injection of Met-Cys mixture may affect the cellular response to oxidative stress.

In terms of mRNA expression, the current study determined that the levels of IGF-I and TLR4 in heart, pectoral muscle, jejunum or liver tissues, were higher in Met-Cys injected group compared to other groups. At the end of incubation period, egg nutrients might be insufficient for broiler embryos, due to the fast growth and protein or energy deficiency that are associated with reduced levels of plasma IGF-I [50]. Kita et al. [51] concluded that dietary addition of arginine, methionine, and cysteine enhanced plasma IGF-I levels and body weight gain in young chicks and added that the early accessibility to critical dietary nutrients would be beneficial. Previous studies also suggested that *In ovo* injection of AA significantly increased growth in broilers [2]. Met-Cys injection has a positive effect on protein synthesis by affecting the expression of genes related to growth. These results agree with those of Del Vesco et al. [52], who concluded that dietary methionine increased IGF-I and GHR mRNA expression in the liver of broilers. In the present study, increased expression of the IGF-I gene in the tissues was observed in Met-Cys injected group, indicating an effective way to increase the embryonic chick weight [30]. Toll-like receptors (TLRs) are extensively studied in rats, but little is known on their role in broiler chickens during heat stress [53]. In the present study, we hypothesized that *In ovo* injection of Met-Cys under heat stress treatment may cause changes in the expression of TLR4 in different organs. As a result of heat stress, cells are able to adjust their metabolism to the changing environmental conditions by modifying their gene expression. These alterations offer sufficient flexibility to preserve homeostasis under stress conditions, maintain their viability and functions, as well as to adapt to long-term changes [54]. Many studies have demonstrated the adverse effect of heat stress treatment on heart, liver, and brain tissues in chickens [13,53]. Heat stress triggers the expression of heat stress-related genes, such as HSP and TLR [53,55]. The TLR4 mediated signal pathway is activated in response to stress, particularly to heat stress [56]. Also, Fukata et al. [57] reported that TLR4 is important for healing the injured intestinal epithelium. Hence, the markedly increased TLR4 mRNA expression levels indicate that the expression of HSP70 was elevated and triggered TLR4 overexpression to protect the intestine from injury by acute heat stress [53].

Heat stress can cause multi-organ system failure, including that of the intestinal tract. The tight junctions between the epithelial cells of the gut lose connectivity, due to oxidation caused by heat stress [58]. Regarding jejunum histomorphometric changes the *In ovo* injection of Met-Cys improved jejunum histomorphometric indexes since the villi height, width, area, crypt depth, histological absorptive surface amplification, and jejunum muscle thickness increased. These results confirmed the hypothesis that *In ovo* injection improves the surface area of villi and crypts depth as indicators of the intestinal developmental and functional status [59,60]. Our results agreed with that of Nazem et al. [15] who found that an injection with methionine increased villus height, width and area and they concluded that injecting methionine can improve intestinal histomorphometric parameters. Moreover, Hou and Tako [61] reported that an increase of a morphometric parameter (villi height, width, area, crypt depth) is expected to improve the digestive and absorptive capabilities of the brush border membrane.

## 5. Conclusions

The results from the current study indicated that *In ovo* injection of Met-Cys mixture after exposure to heat stress during incubation enhanced chick embryonic development, increased thyroid hormone levels, decreased HSP90 level, elevated antioxidant capacity, promoting IGF-I and TLR4 mRNA expression levels and improved jejunum histomorphometric parameters in newly hatched broiler chicks.

## Figures and Tables

**Table 1:** Effect of *In ovo* injection of methionine and cysteine on hatch results and anatomical characteristics of one-day-old broiler chicks.

Items	Treatments			SEM (Standard Error of the Mean)	*p*-Value
	Control	NaCl	Met-Cys		
Hatchability (%)	78	78	80	1.3	0.758
Hatch time (h)	493 ^b^	508 ^a^	505 ^a^	1.6	<0.01
Chick weight (g)	42.12 ^b^	42.67 ^b^	44.66 ^a^	0.38	0.027
Liver (%)	1.75	1.76	1.80	0.03	0.859
Heart (%)	0.57	0.57	0.58	0.01	0.991
Pectoral muscle (%)	0.71	0.72	0.81	0.02	0.146
Small Intestine (%)	1.97	1.99	2.07	0.06	0.779
Left lung (%)	0.30	0.33	0.32	0.01	0.449
Residual yolk (%)	14.24	14.56	14.40	0.44	0.956

^a,b^ Values within a row with different letters differ significantly (*p* < 0.05). Control = Non-injected; NaCl = Saline-injected; Met-Cys = injected with 5.90 mg l-methionine plus 3.40 mg l-cysteine.

**Table 2:** Effect of *In ovo* injection of methionine-cysteine on total protein profiles, uric acid, creatine kinase, thyroid hormones and heat shock protein 90 of newly hatched broiler chicks.

Items	Treatments			SEM	*p*-Value
	Control	NaCl	Met-Cys		
Total Protein (g/L)	16.97	14.92	16.64	0.409	0.124
Albumin (g/L)	9.21	8.88	9.66	0.479	0.804
Globulin (g/L)	7.76	6.04	6.98	0.391	0.231
Albumin/Globulin ratio	1.25	1.74	1.41	0.172	0.506
Uric acid (µmol/L)	46.54 ^b^	46.27 ^b^	50.54 ^a^	1.005	0.002
Creatine kinase (mmol/L)	1.28	1.19	1.28	1.253	0.786
Triiodothyronine (ng/mL)	1.09 ^b^	1.10 ^b^	1.20 ^a^	0.013	0.007
Thyroxin (ng/mL)	58.91 ^b^	52.30 ^b^	72.18 ^a^	1.609	<0.001
Heat shock protein 90 (ng/mL)	6.54^a^	6.52^a^	5.24 ^b^	0.125	0.008

^a,b^ Values within a row with different letters differ significantly (*p* < 0.05). Control = Non-injected; NaCl = Saline-injected; Met-Cys = injected with 5.9 mg l-methionine plus 3.4 mg l-cysteine.

**Table 3:** Effect of *In ovo* injection of methionine-cysteine on total antioxidant capacity and glutathione in serum and tissues of newly hatched broiler chicks.

Items	Treatments			SEM	*p*-Value
	Control	NaCl	Met-Cys		
Serum					
TAOC (U/mL)	8.50 ^b^	8.38 ^b^	11.13 ^a^	0.370	0.023
GSH (µmol/L)	23.54 ^b^	23.45 ^b^	36.93 ^a^	0.579	<0.001
Heart					
TAOC (U/mg protein)	1.51 ^b^	1.65 ^b^	2.37 ^a^	0.051	<0.001
GSH (µmol/g protein)	12.71 ^b^	13.39 ^b^	16.17 ^a^	0.264	<0.001
Pectoral muscle					
TAOC (U/mg protein)	4.19 ^b^	4.55 ^b^	5.80 ^a^	0.214	0.017
GSH (µmol/g protein)	13.36 ^b^	12.46 ^b^	16.08 ^a^	0.206	<0.001
Small intestine					
TAOC (U/mg protein)	9.27 ^b^	8.88 ^b^	10.37 ^a^	0.175	0.009
GSH (µmol/g protein)	19.58 ^b^	19.69 ^b^	21.16 ^a^	0.214	0.015
Liver					
TAOC (U/mg protein)	6.24 ^b^	6.06 ^b^	7.36 ^a^	0.181	0.018
GSH (µmol/g protein)	13.81 ^b^	13.68 ^b^	15.26 ^a^	0.183	0.005
Kidney					
TAOC (U/mg protein)	12.06 ^b^	12.24 ^b^	13.63 ^a^	0.238	0.028
GSH (µmol/g protein)	10.01 ^b^	9.01 ^b^	12.73 ^a^	0.359	0.001

^a,b^ Values within a row with different letters differ significantly (*p* < 0.05). TAOC = Total antioxidant capacity and GSH = Glutathione. Control = Non-injected; NaCl = Saline-injected; Met-Cys = injected with 5.9 mg l-methionine plus 3.4 mg l-cysteine.

**Table 4:** Histomorphometric analysis of jejunum as affected by *In ovo* injection of methionine-cysteine.

Items	Treatments			SEM	*p*-Value
	Control	NaCl	Met-Cys		
Villus height (µm)	298.6 ^b^	314.9 ^b^	335.8 ^a^	9.14	<0.001
Villus width (µm)	53.0 ^b^	52.8 ^b^	60.8 ^a^	2.29	0.002
Villus area (µm)	24,846.5 ^b^	26,104.0 ^b^	32,054.1 ^a^	958.27	<0.001
Crypt depth (µm)	44.9 ^b^	44.9 ^b^	49.7 ^a^	0.90	<0.001
Crypt width (µm)	25.7	24.5	22.8	0.94	0.199
^#^HASA	10.8 ^c^	11.7 ^b^	12.2 ^a^	0.34	0.001
JMT * (µm)	124.6 ^b^	127.5 ^b^	138.0 ^a^	3.41	<0.001

^a,b,c^ Values within a row with different letters differ significantly (*p* < 0.05). * Jejunum muscle thickness, #Histological absorptive surface amplification; Control = Non-injected; NaCl = Saline-injected; Met-Cys = injected with 5.9 mg l-methionine plus 3.4 mg l-cysteine.
